# Influence of Premedication and Dental Anxiety on Anesthetic Efficacy in Patients Undergoing Root Canal for Symptomatic Irreversible Pulpitis in Upper and Lower Molars: A Comparative Study of Articaine and Bupivacaine

**DOI:** 10.3390/dj13050199

**Published:** 2025-04-30

**Authors:** Luis Manteca-Fernández, Cristina Meniz-García, Fernando Fernández-Cáliz, Cristina Barona-Dorado, Juan Santos-Marino, Natalia Martínez-Rodríguez

**Affiliations:** 1 Faculty of Dentistry, University of CEU San Pablo, 28660 Madrid, Spain; luis.mantecafernandez@ceu.es; 2 Department of Dental Clinical Specialties, Faculty of Dentistry, Complutense University of Madrid, 28040 Madrid, Spain; cmenizga@ucm.es (C.M.-G.); fernanfe@ucm.es (F.F.-C.); nataliamartinez@ucm.es (N.M.-R.); 3 Surgical and Implant Therapies in the Oral Cavity Research Group, Complutense University, 28040 Madrid, Spain; 4 Department of Surgery, Faculty of Medicine, University of Salamanca, 37007 Salamanca, Spain; juansantos@usal.es

**Keywords:** irreversible pulpitis, anesthesia, pain

## Abstract

The use of local anesthetics in dentistry is crucial for pain control. Their efficacy may be related to multiple factors, including gender, the clinical status of the patient, anatomical factors, the type of anesthetic, premedication treatment, and the experience of the professional. **Aim:** The objective of this study was to analyze whether premedication or the degree of patient anxiety influences the anesthetic efficacy of 4% articaine with epinephrine 1:100,000 and 0.5% bupivacaine with epinephrine 1:100,000 in patients undergoing root canal treatment for symptomatic irreversible pulpitis in the upper and lower molars. The null hypothesis (H0) of this study was that articaine and bupivacaine would have a similar anesthetic efficacy when used during the treatment of symptomatic irreversible pulpitis of the posterior mandibular and maxillary teeth, independent of non-steroidal anti-inflammatory drugs (NSAIDs) premedication or the patient’s anxiety levels. **Methods:** A total of 140 patients presenting with pulpitis in the upper and lower molars were randomly assigned to one of two anesthetics: articaine or bupivacaine. Before root canal treatment, patients completed the Modified Corah Dental Anxiety Scale (MDAS) and a Visual Analog Scale (VAS) for pain intensity. Inferior alveolar nerve block was performed for the lower molars and buccal infiltration for the upper molars, and the anesthetic efficacy was verified by the Endo Coldspray^®^ test. During the procedure, the patients’ heart rate and oxygen saturation were monitored using a pulse oximeter. The patients reported their pain levels using a VAS twenty-four hours postoperatively. **Results:** High levels of dental anxiety were significantly associated with higher pain scores (*p* = 0.000) for both groups. The hemodynamic changes during treatment remained within normal limits. The need for anesthetic reinforcement was higher in the bupivacaine group (*p* = 0.004). The patients in both groups reported low-intensity postoperative pain, although the pain level was slightly lower in the bupivacaine group. **Conclusions:** The anesthetic efficacy of articaine and bupivacaine in patients with irreversible pulpitis did not appear to be influenced by the degree of anxiety or the intake of AINEs as premedication. The intrinsic anesthetic efficacy was higher for articaine, which required less reinforcement than bupivacaine. Comparing the results obtained when performing buccal infiltration and inferior alveolar nerve block further highlighted the differences between the two anesthetics; these differences were more pronounced in the bupivacaine group, leading to rejection of the null hypothesis proposed at the beginning of the study.

## 1. Introduction

Pulpitis arises from deep caries, trauma, or infections, generating intense pain that may be intermittent or constant. Thermal stimuli, such as cold temperatures, or pressure stimuli, such as mastication, can exacerbate pain, leading to high levels of anxiety in patients [1,2].

Proper pain management during root treatment is crucial, as is the selection of the anesthetic and technique, since inflammation can reduce the efficacy of anesthesia. Modaresi et al. [3] suggested that inflammation significantly decreases the success rates of anesthetics.

The mandibular anesthetic technique typically involves inferior alveolar nerve block (IANB), whereas maxillary anesthesia relies on buccal infiltration techniques. IANB has been associated with a high success rate during the endodontic treatment of mandibular molars [4]; however, this success rate decreases in molars with irreversible pulpitis [5,6]. Afkhami et al. [7] reported that, in a study of 50 patients with irreversible pulpitis in mandibular molars, despite experiencing labial numbness, anesthetic failure occurred in 56% of cases.

The choice of local anesthetic is another critical factor. The most used anesthetic agents in dentistry are mepivacaine, procaine, lidocaine, articaine, and bupivacaine, with the last three being the most extensively documented [8].

Lidocaine has a short onset and, when combined with epinephrine, provides pulpal anesthesia for 60–90 min. Articaine, with its thiophene ring, has greater lipid solubility and a longer duration. Its potency is higher than lidocaine, and its latency period is short, which makes it an appropriate anesthetic for treatments with the presence of pain. Bupivacaine, though slow in onset and high in anesthetic power, provides pulpal anesthesia that lasts 2–3 times longer than lidocaine and is more frequently used in oral surgery [9,10,11,12].

The performance of these anesthetics in cases of irreversible pulpitis that need root treatment is inconsistent [13,14,15]. Some authors, such as Zhang et al. [16], Ghazalgoo et al. [17], and Kanaa et al. [18], reported differences between lidocaine and articaine when they were used during the treatment of pulpitis; meanwhile, others, including Rosenberg et al. [19], Tong et al. [20], and Mittal et al. [21], found no significant differences between them.

Similar discrepancies are present in studies on bupivacaine. Sampaio et al. [12] compared the efficacy of 0.5% bupivacaine and 2% lidocaine in patients with irreversible pulpitis, reporting an 80% success rate for bupivacaine versus a success rate of 62.9% for lidocaine. Conversely, Parirokh et al. [22] reported higher success rates for lidocaine. Gross et al. [23] found that lidocaine was associated with superior results compared to bupivacaine in maxillary infiltrations.

Another aspect to highlight is the use of premedication prior to the treatment of pulpitis, which could achieve greater anesthetic efficacy during root canal treatment. In this sense, there are authors who argue that their prior administration could mask the effects of anesthetics, while other authors, such as Só et al. [24], highlight that the prior administration of drugs such as NSAIDs would improve anesthetic efficacy. The scientific evidence is very varied.

Given these inconsistent findings and the scarcity of studies comparing the use of articaine and bupivacaine in mandibular molars, especially maxillary molars, in performing root canal treatments, we deemed this study necessary. The primary objective of this study was to assess whether AINEs premedication or patient anxiety levels influence the anesthetic efficacy of 4% articaine with epinephrine 1:100,000 and 0.5% bupivacaine with epinephrine 1:100,000 in patients with symptomatic irreversible pulpitis in the upper and lower molars. The null hypothesis (H0) of this study was that articaine and bupivacaine would have a similar anesthetic efficacy when used during the treatment of symptomatic irreversible pulpitis of the posterior mandibular and maxillary teeth, independent of premedication or the patient’s anxiety levels.

## 2. Materials and Methods

### 2.1. Study Design

A randomized clinical study was designed by following the guidelines established in the Declaration of Helsinki and was approved by the Research Ethics Committee of Universidad San Pablo-CEU (C.A. 459/20/49).

### 2.2. Participants

A total of 190 patients from the University Polyclinic of the Faculty of Dentistry at Universidad San Pablo-CEU were intraorally examined for pain in an upper or lower molar. A vitality test was performed on all patients to confirm the presence of irreversible pulpitis using Endo cold spray^®^ (Henry Schein, Melville, NY, USA).

All patients were informed about the possibility of undergoing root canal and provided their informed consent for the procedure; they also consented to the use of their data in this study. The inclusion and exclusion criteria are presented in Table 1. Once these criteria were met, one of the investigators (N.M.-R.) conducted the randomization using opaque envelopes that assigned each patient to one of the study groups: Group 1 (4% articaine with 1:100,000 epinephrine) or Group 2 (0.5% bupivacaine with 1:100,000 epinephrine). Single masking of the patient was performed.

### 2.3. Preoperative Records

Data including patient age, gender, and the molar to be treated (first or second molar, maxillary or mandibular). They were also questioned about previous use of any NSAIDs. To homogenize both groups, patients who reported having been using NSAIDs for more than 24 h were excluded.

Subsequently, patients completed the Modified Corah Dental Anxiety Scale (MDAS), a five-item scale with the following scoring system: (0) Relaxed, not anxious; (1) Slightly anxious; (2) Fairly anxious; (3) Very anxious and uneasy; and (4) Extremely anxious (Table 2). The sum score classification is as follows: ≤9 points indicate no or mild anxiety, 9–12 points indicate moderate anxiety, 13–14 points suggest high anxiety, and >15 points represent severe anxiety levels [25].

Finally, all patients were instructed to record their current pain level on a Visual Analog Scale (VAS, 1–10) [26]. The obtained values were categorized into two groups: baseline pain 6, 7, or 8 (SP: severe pain) and baseline pain 9 or 10 (VSP: very severe pain).

### 2.4. Intraoperative Records: Hemodynamic Changes, Anesthetic Efficacy, and Need for Anesthetic Reinforcement

The anesthetic technique was always performed by the same experienced professional (L.M.-F.), who applied inferior alveolar nerve block for lower molars and buccal infiltration at the vestibular fold for upper molars. The exact time of anesthetic administration completion was recorded. Once patients experienced labial or maxillary gingival numbness, the Endo cold spray^®^ test (Henry Schein, Melville, NY, USA) was repeated. Molars that tested positive were considered anesthetic failures and were administered intraligamentary reinforcement anesthesia. The need for initial anesthetic reinforcement (categorical variable: Yes/No) was recorded. If pain persisted after the first reinforcement, intrapulpal anesthesia was administered.

During the procedure, all patients were monitored using a Beurer^®^ pulse oximeter (Model PO-30 CE, Veszprem, Hungary), with their heart rate and oxygen saturation recorded at four time points: before anesthesia, during anesthetic injection, during pulpal access, and at the end of the treatment.

### 2.5. Postoperative Assessment

Patients were monitored 24 h post-treatment to record the duration of anesthetic effect in minutes. They were previously instructed to note the exact time at which numbness in soft tissues ceased, and their pain levels at 24 h were recorded using a VAS.

### 2.6. Statistical Analysis

Statistical analysis was performed using IBM SPSS Statistics for Windows (Version 27.0, IBM Corp., Armonk, NY, USA).

Descriptive statistics were conducted for the total sample and each group, using mean and standard deviation for quantitative variables and frequencies and percentages for qualitative variables. Normality was assessed using the Kolmogorov–Smirnov and Shapiro–Wilk tests. Qualitative variables were compared using the Chi-square test or Fisher’s exact test, while the Mann–Whitney test was used for quantitative variables due to the rejection of normality in multiple variables. The statistical significance was set at *p* > 0.05.

## 3. Results

### 3.1. Study Population

**Figure 1:**
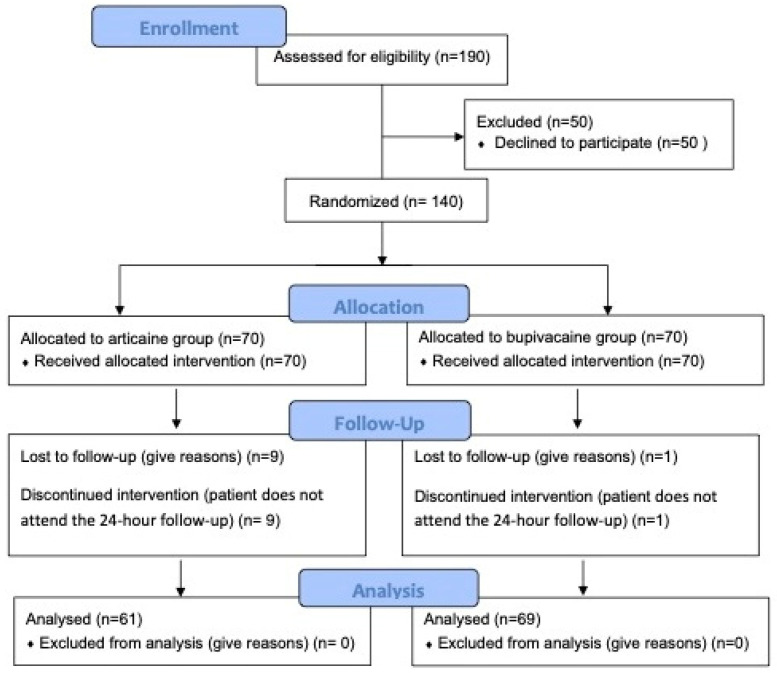
Consort flow diagram.

Out of the 190 patients who attended the CEU San Pablo University Clinic with moderate-severe pain (VAS: 6–10) in one of their maxillary or mandibular molars, 140 agreed to participate in the study after receiving information about its objectives. Ten patients were lost to follow-up, resulting in a final sample of 130 patients (Figure 1). Table 3 presents the initial data collected from the patients at the beginning of the study, showing that the patients’ age and gender distribution were similar in both study groups. Likewise, the distribution of first and second molars was homogeneous according to the maxilla or mandible. No significant differences were found between groups regarding premedication.

### 3.2. Effect of Anxiety and Premedication on Pain

Table 4 shows that the level of anxiety recorded using the Corah questionnaire differed between groups. The articaine group had a higher proportion of patients without anxiety, whereas the bupivacaine group had significantly higher anxiety levels. Regarding its relationship with baseline pain, anxiety significantly influenced the patients’ perception of pain.

Analyzing each group separately, in articaine-anesthetized patients with low anxiety levels, the baseline pain was high; as the patients’ anxiety levels increased, very high levels of pain became more evident. The findings were similar in the bupivacaine group, meaning that there were not significant differences between groups (Table 4).

When evaluating whether premedication influenced the intensity of pain, the results indicated that in non-premedicated patients, the presence of intense and very intense pain was similar in both groups. However, in premedicated patients, very intense pain was most common in the bupivacaine group (*p* > 0.05) (Table 5).

### 3.3. Hemodynamic Changes, Anesthetic Efficacy, and Need for Anesthetic Reinforcement

The pulse oximeter records indicate that, before anesthesia (pre-anesthesia), patients in the articaine group had lower pulse rates than those in the bupivacaine group, a difference that was also present at the end of the treatment and was statistically significant. During anesthetic injection and chamber opening, the pulse rates were similar for both groups in the other controls.

Regarding oxygen saturation, although the values remained within normal limits in both groups, statistically significant differences were found during anesthetic injection, chamber opening, and at the end of the treatment, with higher values observed in the bupivacaine group (Table 6).

### 3.4. Effect of Anxiety, Premedication, and Administration Technique on Anesthetic Efficacy

The overall anesthetic efficacy of articaine was clearly superior compared to bupivacaine. The need for additional anesthesia was also reduced in the articaine group, with only 26.71% of patients in this group requiring supplementary intrapulpal anesthesia following intraligamentary anesthesia; meanwhile, in the bupivacaine group, intrapulpal anesthesia was required in 45.09% of cases (Table 6).

Regarding the potential influence of premedication intake on the need for additional anesthesia, the need for reinforcement was similar in both groups of patients who had not taken premedication: 59.09% for articaine compared to 62.07% for bupivacaine. Conversely, among premedicated patients, the need for reinforcement was significantly higher in the bupivacaine group, at 82.5%, compared to 56.41% in the articaine group (Table 6).

Although anxiety levels were statistically different between the groups, when analyzing whether the degree of anxiety influenced the efficacy of the anesthetic, the results for the articaine group (*p* = 0.307) and the bupivacaine group (*p* = 0.157) were not statistically significant.

Finally, regarding the two techniques employed—IANB for lower molars and buccal infiltration for upper molars—the results indicated that, in the articaine group, the efficacy of both techniques was very similar; this is reflected in the frequency with which additional anesthesia was required. However, in the bupivacaine group, the efficacy was higher for upper molars, which required less additional anesthesia than the lower molars (Table 7).

### 3.5. Anesthetic Duration and Postoperative Pain

The duration of the anesthetic effect was significantly higher for patients anesthetized with bupivacaine. Additionally, patients in the bupivacaine group reported less pain at 24 h compared to those in the articaine group. However, it is important to note that the pain level was categorized as mild (Table 8).

## 4. Discussion

The primary objective of this study was to analyze the anesthetic efficacy of articaine and bupivacaine in the upper and lower molars of patients with irreversible pulpitis.

The use of local anesthetics in dentistry is crucial for pain control. Their efficacy may be related to multiple factors, including gender, the clinical status of the patient, anatomical factors, the type of anesthetic, and the experience of the professional [27].

We did not find any difference in patients’ perception of pain based on gender. This is inconsistent with multiple studies suggesting that women experience more intense orofacial pain [28,29,30]. However, when analyzing patients with pulpitis and root canal treatments, Segura-Egea et al. [31] and Nusstein et al. [32] did not find differences between men and women. Thus, our findings support the notion that, while general orofacial pain levels may differ between genders, pain associated with root canal treatment for pulpitis does not.

Another relevant aspect was the influence of anxiety levels on the patients’ pain response and, consequently, the efficacy of the anesthetic used; this is particularly important considering that a significant proportion of the patient population experienced fear and anxiety regarding dental treatments. Our results demonstrate that the intensity of the pain and anxiety experienced by patients are directly related. Dou et al. [33] also observed this relationship, highlighting that a high percentage of individuals with irreversible pulpitis suffer from dental anxiety. Yu et al. [34] confirmed this anxiety–pain association, emphasizing that age may strongly correlate with dental anxiety. Other researchers have evaluated whether anxiety levels differ based on anesthetic techniques, concluding that technique does not appear to influence anxiety levels [35,36,37,38].

Similarly, anxiety and pain levels, along with the pharmacological actions of anesthetics, could contribute to hemodynamic changes. In our study, the patients’ pulse rates were recorded, showing increases during anesthetic injection and chamber opening in both groups. Although patients in the bupivacaine group exhibited significantly higher pulse rates at the end of the procedure, these values should be interpreted as normal. Regarding oxygen saturation, the values remained normal, and no significant variations were observed. Other authors have also analyzed these hemodynamic changes. Abu-Mostafa et al. [39] found that during dental extractions performed with 2% lidocaine with 1:80,000 epinephrine, 4% articaine with 1:100,000 epinephrine, and 4% articaine with 1:200,000 epinephrine, no significant differences in patients’ hemodynamic records were observed. These findings were corroborated by Pereira et al. [40], who compared 1:100,000 and 1:200,000 articaine in patients with mandibular molar pulpitis and found no significant changes. Moaddabi et al. [41] concluded that articaine is as safe as lidocaine, as it does not cause significant hemodynamic changes during maxillary infiltrative anesthesia.

Bupivacaine is a potent and long-lasting anesthetic with a known cardiotoxic effect [42,43,44]. Consequently, its use in dentistry has been primarily restricted to oral surgery, particularly in third molar extractions, and presents mixed results. Sancho-Puchades et al. [45] compared 0.5% bupivacaine with 4% articaine and reported greater hemodynamic changes with articaine. Conversely, Gregorio et al. [46] observed a greater increase in blood pressure in patients anesthetized with bupivacaine compared to articaine. This variability is further illustrated by Bagatin et al. [47], who suggested that bupivacaine’s anesthetic potency reduces the hemodynamic changes caused by adrenaline, whereas Gonca et al. [48] reported that epinephrine reduces cardiotoxic effects in rats.

The results reported in the scientific literature vary considerably across studies. Sülek et al. [49] found that articaine was effective in approximately 70% of patients with pulpitis in the lower premolars. In studies comparing articaine and lidocaine, Hassan et al. [50] reported that during the preparation of the access cavity, lidocaine had a success rate of 93%, while articaine exhibited a slightly higher success rate of 97%. Conversely, Daneshvar et al. [51] concluded that lidocaine is more effective than articaine in patients with mandibular pulpitis. Jouhar et al. [52] found an efficacy rate of 76.7% for bupivacaine compared to 40% for lidocaine. A systematic review and meta-analysis by Larocca de Geus et al. [53] analyzed the success of IANB with different anesthetics, concluding that, in cases of irreversible pulpitis, the probability of success was 73% for articaine, 57% for prilocaine, 55% for mepivacaine, 53% for bupivacaine, and 12% for lidocaine. These findings contrast with the results of this study, which reported an efficacy of 42.6% for articaine and 26.08% for bupivacaine. However, these results are more consistent with the double-blind clinical trial conducted by Aggarwal et al. [54], which reported success rates of 23% for 2% lidocaine, 33% for 4% articaine, and 17% for 0.5% bupivacaine. One possible explanation for these discrepancies is that our study did not exclude patients taking medication; in addition, the inclusion of maxillary molars could have influenced the results, as prior studies primarily focused on mandibular molars. Regarding the first point, some studies assessing the efficacy of anesthetic in patients with irreversible pulpitis exclude individuals with a history of prior medication intake to avoid alterations in their perception of pain [50,55]. However, scientific evidence suggests that the pre-administration of medications such as meloxicam, ketorolac, dexamethasone, and ibuprofen enhances the efficacy of anesthetics in patients with irreversible pulpitis [56,57,58].

The data collected in this study were relatively similar between groups: 59.42% of patients in the bupivacaine group reported taking premedication drugs compared to 62.29% in the articaine group. This medication intake does not appear to have significantly influenced the study, as premedicated patients in the bupivacaine group required reinforcement in 82.50% of cases, compared to 56.41% in the articaine group.

When analyzing differences between the two anesthetic techniques used—buccal infiltration in the maxilla and IANB in the mandible—our results determined that the anesthetic efficacy was slightly higher for articaine during buccal infiltration (reinforcement: 56.04%) than IANB). In contrast, these differences were more pronounced in the bupivacaine group. The differences in efficacy have been analyzed in studies primarily comparing articaine and lidocaine. Atasoy et al. [59], Afkhami et al. [60], and Nogueira et al. [61] reported that the rate of anesthetic efficacy in upper molars with irreversible pulpitis Conversely, Srinivasan et al. [62] found that articaine achieved 100% efficacy, in contrast to 80% for lidocaine. Hosseini et al. [63] reported a 66.7% efficacy for articaine and 56.52% for lidocaine, similar to the results obtained by Syed et al. [64]. A recent study by Kumar et al. [65] supported these findings, reporting an efficacy rate of 74% for articaine and a rate of 69% for lidocaine. Another systematic review by Almadhoon et al. [66] concluded that buccal infiltration with articaine produced comparable results to IANB; however, the authors stated that these findings should be interpreted with caution due to the limited number of included studies.

Lastly, the postoperative records collected 24 h after treatment showed a significant reduction in pain compared to the baseline pain levels. The values obtained can be considered mild, with slightly lower levels in patients anesthetized with bupivacaine. This may be interpreted because of both the efficacy of the root canal treatment itself and a residual analgesic effect of the anesthetics, as suggested by some studies. Talebzadeh et al. [67], in a clinical trial involving 96 patients who reported their pain using VAS (1–100), found the patients’ scores ranged between 26 and 34 four hours after root canal treatment; this gradually decreased to values between 8 and 14 at 24 h. Regarding the anesthetics used, Parirokh et al. [22] found that patients anesthetized with bupivacaine for irreversible pulpitis in the mandibular molars experienced significantly less postoperative pain and required fewer analgesics compared to those anesthetized with lidocaine. These results align with those reported by Miroshnychenko et al. [68], who, in a systematic review comparing long-acting and short-acting local anesthetics, suggested that bupivacaine may reduce the need for rescue analgesia without causing additional adverse effects; however, the certainty of this evidence was low. A new systematic review by Alhilou et al. [69] concluded that the randomized controlled trials available report varying results, justifying the need for additional well-designed studies to determine the most effective treatments for reducing pain in patients with irreversible pulpitis.

There were some limitations to this study. For example, we did not consider anatomical variations in innervation that could have affected the response of patients to anesthetic. Additionally, patients’ pain perceptions and thresholds are subjective and often inconsistent. Moreover, in this study, anxiety levels were higher in the bupivacaine group, which could explain some of the differences found. The high percentage of additional anesthetic requirements should not necessarily be interpreted as questioning the intrinsic efficacy of these anesthetics. A future study comparing patients with mild pain versus those with intense pain could further validate the excellent results that both articaine and bupivacaine achieve under physiological conditions.

## 5. Conclusions

The anesthetic efficacy of articaine and bupivacaine in patients undergoing root canal for irreversible pulpitis does not appear to be influenced by anxiety levels or the intake of premedication. The intrinsic efficacy was greater for articaine, which required less reinforcement than bupivacaine. When comparing the results of the buccal infiltration technique and IANB, differences between the anesthetics were observed once again, with more pronounced differences being observed in the bupivacaine group; this led to the rejection of the null hypothesis proposed at the beginning of the study.

## Figures and Tables

**Table 1:** The inclusion and exclusion criteria of the study.

Inclusion Criteria	Exclusion Criteria
1. Male and female patients over 18 years of age.	1. Patients who did not wish to participate in the study.
2. ASA I or II patients.	2. Patients who have self-medicated with non-steroidal anti-inflammatories for more than 24 h.
3. Patients requiring endodontic treatment for an irreversible pulpitis-affected molar.	3. Patients who did not fully complete any of the questionnaires.
4. Baseline pain > 6 on a Visual Analog Scale (VAS).	4. Patients who did not attend the 24-h follow-up.
	5. Patients for whom endodontic treatment could not be completed in a single session.

**Table 2:** Modified Dental Anxiety Scale (MDAS): a five-item questionnaire.

Corah Anxiety Questionnaire
If you were going to the dentist tomorrow for a checkup, how would you feel about it?
(1) Relaxed, not anxious. (2) Slightly anxious. (3) Fairly anxious. (4) Very anxious and uneasy. (5) Extremely anxious (sweating, tachycardia, feeling severely unwell).
2. While waiting for your turn in the dentist’s waiting room, how do you feel?
(1) Relaxed, not anxious. (2) Slightly anxious. (3) Fairly anxious. (4) Very anxious and uneasy. (5) Extremely anxious (sweating, tachycardia, feeling severely unwell).
3. When sitting in the dentist’s chair while the dentist prepares the drill to start working on your teeth, how do you feel?
(1) Relaxed, not anxious. (2) Slightly anxious. (3) Fairly anxious. (4) Very anxious and uneasy. (5) Extremely anxious (sweating, tachycardia, feeling severely unwell).
4. Imagine you are in the dentist’s chair for a cleaning. While waiting, the dentist or hygienist pulls out the instruments used to scrape your teeth around the gums. How do you feel?
(1) Relaxed, not anxious. (2) Slightly anxious. (3) Fairly anxious. (4) Very anxious and uneasy. (5) Extremely anxious (sweating, tachycardia, feeling severely unwell).
5. If you were to receive a local anesthetic injection for your dental treatment, how would you feel?
(1) Relaxed, not anxious. (2) Slightly anxious. (3) Fairly anxious. (4) Very anxious and uneasy. (5) Extremely anxious (sweating, tachycardia, feeling severely unwell).

**Table 3:** Characteristics of patients, according to age, gender, and affected molar.

	Articaine (n = 61 Patients)	Bupivacaine (n = 69 Patients)	*p*-Value
**Age** (years)	49.74 ± 17.07	47.88 ± 18.36	0.55
**Gender**			
Men	31	33	
Women	30	36	0.73
**Molar**			
Maxillary first molar	15	19	
Maxillary second molar	16	19	0.88
Mandibular first molar	17	15	
Mandibular second molar	13	16	
**Premedication**			
Yes	16	26	0.16
No	45	43	

**Table 4:** Dental anxiety expressed in levels and percentages. Baseline pain levels (SP: severe pain, VSP: very severe pain) and percentage.

	Dental Anxiety	Baseline Pain Levels
**Articaine**	**<9**: 32.8%	SP: 70% VSP: 30%
	**9–12**: 54.1%	SP: 42.4% VSP: 54.5%
	**13–14**: 6.6%	SP: 50% VSP: 50%
	**>14**: 6.6%	SP: 0% VSP: 100%
**Bupivacaine**	**<9**: 1.4%	SP: 100% VSP: 0%
	**9–12**: 53.6%	SP: 48.6% VSP: 51.3%
	**13–14**: 30.4%	SP: 23.8% VSP: 76.1%
	**>14**: 14.5%	SP: 40% VSP: 60%

**Table 5:** Influence of premedication on pain levels (SP: severe pain, VSP: very severe pain).

	**No premedication** 45 patients	**Premedication** 16 patients
**Articaine**	SP: 59.09% VSP: 40.9%	SP: 56.4% VSP: 43.5%
	**No premedication** 43 patients	**Premedication** 26 patients
**Bupivacaine**	SP: 62.07% VSP: 37.9%	SP: 82.5% VSP: 17.5%
	**9–12**: 53.6%	SP: 48.6% VSP: 51.3%
	**13–14**: 30.4%	SP: 23.8% VSP: 76.1%
	**>14**: 14.5%	SP: 40% VSP: 60%

**Table 6:** Record of pulse and oxygen saturation.

	Articaine (n = 61)	Bupivacaine (n = 69)	*p*-Value
**Beats per minute**			
Pre-anesthesia	71.08 ± 6.99	73.88 ± 7.94	0.04
Anesthetic injection	73.59 ± 7.39	74.80 ± 8.31	0.39
Chamber opening	75.36 ± 8.17	75.23 ± 7.89	0.93
Treatment completion	72.21 ± 6.72	75.35 ± 9.35	0.03
**Oxygen saturation**			
Pre-anesthesia	97.89 ± 1.70	98.29 ± 4.01	0.38
Anesthetic injection	97.84 ± 1.69	98.59 ± 1.31	0.00
Chamber opening	97.90 ± 1.65	98.83 ± 1.16	0.00
Treatment completion	97.82 ± 1.68	98.86 ± 1.19	0.00

**Table 7:** Anesthetic efficacy and the need for reinforcement.

	Articaine	Bupivacaine	*p*-Value
Anesthetic efficacy	26 (42.62%)	18 (26.08%)	0.04
Anesthetic reinforcement	35 (57.4%)	51 (73.91%)	0.00
Intraligamentary technique	26 (74.28%)	28 (54.91%)	0.06
Intrapulpal technique	9 (25.71%)	23 (45.09%)	0.06
No premedication	16 (59.09%)	26 (62.07%)	0.99
With premedication	45 (56.41%)	43 (82.50%)	0.01
Maxillary molars	31 (56.04%)	38 (63.94%)	0.14
Mandibular molars	30 (58.71%)	31 (86.67%)	0.11

**Table 8:** Duration of anesthetic effect and post-treatment pain recording.

	Articaine (n = 61)	Bupivacaine (n = 69)	*p*-Value
**Anesthetic duration in minutes**	159.26 ± 35.69	264.35 ± 26.70	0.00
**Pain at 24 h (VAS)**	2.61 ± 1.48	1.93 ± 1.34	0.00

## Data Availability

The data presented in this study are available on request from the corresponding author. The data are not publicly available due to privacy reasons.

## References

[1] Zanini M., Meyer E., Simon S. (2017). Pulp Inflammation Diagnosis from Clinical to Inflammatory Mediators: A Systematic Review. J. Endod..

[2] Erdogan O., Malek M., Gibbs J.L. (2021). Associations between Pain Severity, Clinical Findings, and Endodontic Disease: A Cross-Sectional Study. J. Endod..

[3] Modaresi J., Dianat O., Soluti A. (2008). Effect of pulp inflammation on nerve impulse quality with or without anesthesia. J. Endod..

[4] Hung P.C., Chang H.H., Yang P.J., Kuo Y.S., Lan W.H., Lin C.P. (2006). Comparison of the Gow-Gates mandibular block and inferior alveolar nerve block using a standardized protocol. J. Formos. Med. Assoc..

[5] Kanaa M.D., Whitworth J.M., Meechan J.G. (2012). A prospective randomized trial of different supplementary local anesthetic techniques after failure of inferior alveolar nerve block in patients with irreversible pulpitis in mandibular teeth. J. Endod..

[6] Yadav S. (2015). Anesthetic success of supplemental infiltration in mandibular molars with irreversible pulpitis: A systematic review. J. Conserv. Dent..

[7] Afkhami F., Ghabraei S., Hashemi N., Peters O.A. (2025). Evaluation of Cold and Electric Pulp Tests for Assessing the Success of Inferior Alveolar Nerve Block for Mandibular First Molars Diagnosed with Symptomatic Irreversible Pulpitis. J. Endod..

[8] St George G., Morgan A., Meechan J., Moles D.R., Needleman I., Ng Y.L., Petrie A. (2018). Injectable local anaesthetic agents for dental anaesthesia. Cochrane Database Syst. Rev..

[9] Hargreaves K.M., Keiser K. (2002). Local anesthetic failure in endodontics: Mechanisms and management. Endod. Top..

[10] Winther J.E., Patirupanusara B. (1974). Evaluation of carticaine—A new local analgesic. Int. J. Oral. Surg..

[11] Haas D.A., Harper D.G., Saso M.A., Young E.R. (1990). Comparison of articaine and prilocaine anesthesia by infiltration in maxillary and mandibular arches. Anesth. Prog..

[12] Sampaio R.M., Carnaval T.G., Lanfredi C.B., Horliana A.C., Rocha R.G., Tortamano I.P. (2012). Comparison of the anesthetic efficacy between bupivacaine and lidocaine in patients with irreversible pulpitis of mandibular molar. J. Endod..

[13] Claffey E., Reader A., Nusstein J., Beck M., Weaver J. (2004). Anesthetic efficacy of articaine for inferior alveolar nerve blocks in patients with irreversible pulpitis. J. Endod..

[14] Tortamano I.P., Siviero M., Costa C.G., Buscariolo I.A., Armonia P.L. (2009). A comparison of the anesthetic efficacy of articaine and lidocaine in patients with irreversible pulpitis. J. Endod..

[15] Mikesell P., Nusstein J., Reader A., Beck M., Weaver J. (2005). A comparison of articaine and lidocaine for inferior alveolar nerve blocks. J. Endod..

[16] Zhang A., Tang H., Liu S., Ma C., Ma S., Zhao H. (2019). Anesthetic efficiency of articaine versus lidocaine in the extraction of lower third molars: A meta-analysis and systematic review. J. Oral. Maxillofac. Surg..

[17] Ghazalgoo A., Saatchi M., Khazaei S., Shadmehr E. (2018). The effect of using articaine versus lidocaine for inferior alveolar nerve block on pain after root canal treatment: A Prospective, Randomized clinical study. Dent. Hypotheses.

[18] Kanaa M.D., Whitworth J.M., Corbett I.P., Meechan J.G. (2006). Articaine and lidocaine mandibular buccal infiltration anesthesia: A prospective randomized double-blind cross-over study. J. Endod..

[19] Rosenberg P.A., Amin K.G., Zibari Y., Lin L.M. (2007). Comparison of 4% articaine with 1: 100,000 epinephrine and 2% lidocaine with 1: 100,000 epinephrine when used as a supplemental anesthetic. J. Endod..

[20] Tong H.J., Alzahrani F.S., Sim Y.F., Tahmassebi J.F., Duggal M. (2018). Anaesthetic efficacy of articaine versus lidocaine in children’s dentistry: A systematic review and meta-analysis. Int. J. Paediatr. Dent..

[21] Mittal J., Kaur G., Mann H.S., Narang S., Kamra M., Kapoor S., Sindhi M., Kataria R. (2018). Comparative Study of the Efficacy of 4% Articaine vs. 2% Lidocaine in Surgical Removal of Bilaterally Impacted Mandibular Third Molars. J. Contemp. Dent. Pract..

[22] Parirokh M., Yosefi M.H., Nakhaee N., Manochehrifar H., Abbott P.V., RezaForghani F. (2012). Effect of bupivacaine on postoperative pain for inferior alveolarnerve block anesthesia after single-visit root canal treatment in teethwith irreversible pulpitis. J. Endod..

[23] Gross R., McCartney M., Reader A., Beck M. (2007). A prospective, randomized, double-blind comparison of bupivacaine and lidocaine for maxillary infiltrations. J. Endod..

[24] Só G.B., Silva I.A., Weissheimer T., Lenzi T.L., Só M.V.R., da Rosa R.A. (2023). Do NSAIDs used prior to standard inferior alveolar nerve blocks improve the analgesia of mandibular molars with irreversible pulpitis? An umbrella review. Clin. Oral. Investig..

[25] Carrillo-Diaz M., Crego A., Armfield J.M., Romero M. (2012). Adaptation and Psychometric Properties of the Spanish Version of the Index of Dental Anxiety and Fear (IDAF-4C+). Oral. Health Prev. Dent..

[26] Heller G.Z., Manuguerra M., Chow R. (2016). How to analyze the Visual Analogue Scale: Myths, truths and clinical relevance. Scand. J. Pain..

[27] Poorni S., Veniashok B., Senthilkumar A.D., Indira R., Ramachandran S. (2011). Anestheticefficacyoffourpercentarticaineforpulpalanesthesiabyusing inferior alveolar nerve block and buccal infiltration techniques in patients with irreversible pulpitis: A prospective randomized double-blind clinical trial. J. Endod..

[28] Sultan H., Pervez H., Maqsood S., Zeeshan W.S. (2023). Evaluation of pain experienced by orthodontic patients following elastomeric separator insertion: A cross-sectional study. Korean J. Orthod..

[29] Sharma S., Lövgren A., Åkerman S., Nilsson P.M., Axtelius B., List T., Henrikson B.H. (2021). Prevalence of Facial Pain and Headache in Sweden. J. Oral. Facial Pain. Headache.

[30] Häggman-Henrikson B., Liv P., Ilgunas A., Visscher C.M., Lobbezoo F., Durham J., Lövgren A. (2020). Increasing gender differences in the prevalence and chronification of orofacial pain in the population. Pain.

[31] Segura-Egea J.J., Cisneros-Cabello R., Llamas-Carreras J.M., Velasco-Ortega E. (2009). Pain associated with root canal treatment. Int. Endod. J..

[32] Nusstein J.M., Beck M. (2003). Comparison of preoperative pain and medication use in emergency patients presenting with irreversible pulpitis or teeth with necrotic pulps. Oral. Surg. Oral. Med. Oral. Pathol. Oral. Radiol. Endod..

[33] Dou L., Vanschaayk M.M., Zhang Y., Fu X., Ji P., Yang D. (2018). The prevalence of dental anxiety and its association with pain and other variables among adult patients with irreversible pulpitis. BMC Oral. Health.

[34] Yu J., Jiang R., Nie E.M., Zhang C.Y., Li X. (2021). The Prevalence of Dental Anxiety Associated with Pain among Chinese Adult Patients in Guangzhou. Pain. Res. Manag..

[35] de Camargo Smolarek P., da Silva L.S., Martins P.R.D., da Cruz Hartman K., Bortoluzzi M.C., Chibinski A.C.R. (2021). The influence of distinct techniques of local dental anesthesia in 9- to 12-year-old children: Randomized clinical trial on pain and anxiety. Clin. Oral. Investig..

[36] Smolarek P.C., da Silva L.S., Martins P.R.D., Hartman K.D.C., Bortoluzzi M.C., Chibinski A.C.R. (2020). Evaluation of pain, disruptive behaviour and anxiety in children aging 5–8 years old undergoing different modalities of local anaesthetic injection for dental treatment: A randomised clinical trial. Acta Odontol. Scand..

[37] Anil Ö., Keskin G. (2024). Comparison of computer controlled local anesthetic delivery and traditional injection regarding disruptive behaviour, pain, anxiety and biochemical parameters: A randomized controlled trial. J. Clin. Pediatr. Dent..

[38] Kuscu O.O., Akyuz S. (2008). Is it the injection device or the anxiety experienced that causes pain during dental local anaesthesia?. Int. J. Paediatr. Dent..

[39] Abu-Mostafa N., Al-Showaikhat F., Al-Shubbar F., Al-Zawad K., Al-Zawad F. (2015). Hemodynamic changes following injection of local anesthetics with different concentrations of epinephrine during simple tooth extraction: A prospective randomized clinical trial. J. Clin. Exp. Dent..

[40] Pereira L.A., Groppo F.C., Bergamaschi C.d.C., Meechan J.G., Ramacciato J.C., Motta R.H., Ranali J. (2013). Articaine (4%) with epinephrine (1:100,000 or 1:200,000) in intraosseous injections in symptomatic irreversible pulpitis of mandibular molars: Anesthetic efficacy and cardiovascular effects. Oral. Surg. Oral. Med. Oral. Pathol. Oral. Radiol..

[41] Moaddabi A., Soltani P., Zamanzadeh M., Nosrati K., Mollamirzaei M., Cernera M., Spagnuolo G. (2021). Comparison of the Effects of Articaine and Lidocaine Anesthetics on Blood Pressure after Maxillary Infiltration Technique: A Triple-Blind Randomized Clinical Trial. Int. J. Dent..

[42] Kim J.T., Yang S.M., Lee K.H. (2013). The effects of insulin-glucose-potassium (IGK) pretreatment on bupivacaine cardiotoxicity. Korean J. Anesthesiol..

[43] Pişkin Ö., Ayoğlu H. (2018). Effects of Remifentanil Pretreatment on Bupivacaine Cardiotoxicity in Rats. Cardiovasc. Toxicol..

[44] Luo M., Yun X., Chen C., Bao N., Feng X., Pan L., Jin Z., Wu C., Wang X., Papadimos T.J. (2016). Giving Priority to Lipid Administration Can Reduce Lung Injury Caused by Epinephrine in Bupivacaine-Induced Cardiac Depression. Reg. Anesth. Pain. Med..

[45] Sancho-Puchades M., Vílchez-Pérez M.Á., Valmaseda-Castellón E., Paredes-García J., Berini-Aytés L., Gay-Escoda C. (2012). Bupivacaine 0.5% versus articaine 4% for the removal of lower third molars. A crossover randomized controlled trial. Med. Oral. Patol. Oral. Cir. Bucal..

[46] Gregorio L.V., Giglio F.P., Sakai V.T., Modena K.C., Colombini B.L., Calvo A.M., Sipert C.R., Dionísio T.J., Lauris J.R., Faria F.A. (2008). A comparison of the clinical anesthetic efficacy of 4% articaine and 0.5% bupivacaine (both with 1:200,000 epinephrine) for lower third molar removal. Oral. Surg. Oral. Med. Oral. Pathol. Oral. Radiol. Endod..

[47] Bagatin T., Škrtić M., Šakić L., Bagatin D., Šakić K., Deutsch J., Šklebar I. (2022). Hemodynamic function in comparison of two types of local anesthesia with vasoconstrictor in day surgery: Retrospective study. Acta Clin. Croat..

[48] Gonca E., Çatlı D. (2018). The Effects of Lidocaine with Epinephrine on Bupivacaine-Induced Cardiotoxicity. Turk. J. Anaesthesiol. Reanim..

[49] Sülek T., Dumani A., Küden C., Kussever H., Yoldas O. (2025). Anesthetic effectiveness of mental/incisive nerve block versus inferior alveolar nerve block in mandibular first and second premolars with symptomatic irreversible pulpitis: A randomized clinical trial. J. Endod..

[50] Hassan S., Ahmed A., Saqib W., Abulhamael A.M., Habib S.R., Javed M.Q. (2023). Comparison of Efficacy of Lidocaine and Articaine as Inferior Alveolar Nerve Blocking Agents in Patients with Symptomatic Irreversible Pulpitis: Randomized Controlled Trial. Medicina.

[51] Daneshvar S.H., Dorani D., Daneshvar M.M. (2021). Comparison of anaesthetic efficacy of 4% articaine buccal infiltration versus 2% lidocaine inferior alveolar nerve block for pulpotomy in mandibular primary second molars. J. Indian. Soc. Pedod. Prev. Dent..

[52] Jouhar R., Ahmed M.A., Ghani B. (2020). Determination of Anesthetic Efficacy of Lidocaine Versus Bupivacaine in Single Visit Root Canal Treatment. Eur. Endod. J..

[53] Larocca de Geus J., Nogueira da Costa J.K., Wambier L.M., Maran B.M., Loguercio A.D., Reis A. (2020). Different anesthetics on the efficacy of inferior alveolar nerve block in patients with irreversible pulpitis: A network systematic review and meta-analysis. J. Am. Dent. Assoc..

[54] Aggarwal V., Singla M., Miglani S. (2017). Comparative Evaluation of Anesthetic Efficacy of 2% Lidocaine, 4% Articaine, and 0.5% Bupivacaine on Inferior Alveolar Nerve Block in Patients with Symptomatic Irreversible Pulpitis: A Prospective, Randomized, Double-blind Clinical Trial. J. Oral. Facial Pain Headache.

[55] Vatankhah M., Zargar N., Naseri M., Sadeghi S., Baghban A.A., Dianat O., Nusstein J.M. (2023). Primary and supplementary anesthetic efficacy of a modified two-step buccal infiltration of 4% articaine in mandibular molars with symptomatic irreversible pulpitis: A randomized clinical trial. Clin. Oral. Investig..

[56] de Oliveira J.P., de Alencar A.H.G., Estrela C.B., Decurcio D.A., Estrela C.R.A., Estrela C. (2024). Comparative effectiveness of preemptive administration of ibuprofen and ibuprofen-arginine on the anesthetic success of inferior alveolar nerve block in teeth with symptomatic irreversible pulpitis—A double-blind randomized clinical trial. Clin. Oral. Investig..

[57] Rodrigues G.A., Hizatugu R., Bronzato J.D., de-Jesus-Soares A., Frozoni M. (2024). Effect of preemptive use of a nonsteroidal anti-inflammatory drug and a corticosteroid on the efficacy of inferior alveolar nerve blockade and postoperative pain control in endodontic treatment of molars with symptomatic pulpitis: A randomized double-blind placebo-controlled clinical trial. Int. Endod. J..

[58] Elnaghy A.M., Elshazli A.H., Elsaka S.E. (2023). Effectiveness of oral premedication of meloxicam, ketorolac, dexamethasone, and ibuprofen on the success rate of inferior alveolar nerve block in patients with symptomatic irreversible pulpitis: A prospective, double-blind, randomized controlled trial. Quintessence Int..

[59] Atasoy Ulusoy Ö.İ., Alaçam T. (2014). Efficacy of single buccal infiltrations for maxillary first molars in patients with irreversible pulpitis: A randomized controlled clinical trial. Int. Endod. J..

[60] Afkhami F., Rostami G., Peters O.A., Kamalian F. (2023). Pulpal anesthesia of maxillary first molars using 4% articaine infiltration in patients with symptomatic irreversible pulpitis: A randomized controlled clinical trial. Clin. Oral. Investig..

[61] Nogueira A.P.A., Ferreira M.C., Maia C.C.R., Gonçalves B.L.L., Filho E.M., Costa C.P., Gavini G., Grazziotin-Soares R., Carvalho C.N. (2024). Efficacy of articaine anesthesia with needle-free/Comfort-in method and conventional needle injection in dental patients with irreversible pulpitis: A randomized clinical trial. Clin. Oral. Investig..

[62] Srinivasan N., Kavitha M., Loganathan C.S., Padmini G. (2009). Comparison of anesthetic efficacy of 4% articaine and 2% lidocaine for maxillary buccal infiltration in patients with irreversible pulpitis. Oral. Surg. Oral. Med. Oral. Pathol. Oral. Radiol. Endod..

[63] Hosseini H.R., Parirokh M., Nakhaee N., VAbbott P., Samani S. (2016). Efficacy of Articaine and Lidocaine for Buccal Infiltration of First Maxillary Molars with Symptomatic Irreversible Pulpitis: A Randomized Double-blinded Clinical Trial. Iran Endod. J..

[64] Syed G.A., Mulay S.A. (2022). Comparative Evaluation of Anesthetic Efficacy of 4% Articaine and 2% Lidocaine for Buccal Infiltration in Adult Patients with Irreversible Pulpitis of Maxillary First Molar: A Prospective Randomized Study. Contemp. Clin. Dent..

[65] Kumar V., Chawla A., Priya H., Sachdeva A., Sharma S., Kumar V., Logani A. (2024). Comparative evaluation of full and partial pulpotomy in permanent teeth with irreversible pulpitis: A systematic review and meta-analysis. Aust. Endod. J..

[66] Almadhoon H.W., Abuiriban R.W., Almassri H., Al-Hamed F.S. (2022). Efficacy of 4% articaine buccal infiltration versus inferior alveolar nerve block for mandibular molars with symptomatic irreversible pulpitis: A systematic review and meta-analysis. J. Evid. Based Dent. Pract..

[67] Talebzadeh B., Nezafati S., Rahimi S., Shahi S., Lotfi M., Ghasemi N. (2016). Comparison of Manual and Rotary Instrumentation on Postoperative Pain in Teeth with Asymptomatic Irreversible Pulpitis: A Randomized Clinical Trial. Iran Endod. J..

[68] Miroshnychenko A., Ibrahim S., Azab M., Roldan Y., Diaz Martinez J.P., Tamilselvan D., He L., Urquhart O., Tampi M., Polk D.E. (2023). Injectable and topical local anesthetics for acute dental pain: 2 systematic reviews. J. Am. Dent. Assoc..

[69] Alhilou A.M., Al-Moraissi E.A., Bakhsh A., Christidis N., Näsman P. (2023). Pain after emergency treatments of symptomatic irreversible pulpitis and symptomatic apical periodontitis in the permanent dentition: A systematic review of randomized clinical trials. Front. Oral. Health.

